# HAVCR1 Affects the MEK/ERK Pathway in Gastric Adenocarcinomas and Influences Tumor Progression and Patient Outcome

**DOI:** 10.1155/2019/6746970

**Published:** 2019-12-02

**Authors:** Ji Xue, Ying Li, Jianfeng Yi, Hong Jiang

**Affiliations:** ^1^Department of Traditional Chinese Medicine, The Second Hospital of Jilin University, No. 218 Nanguan District, Changchun, Jilin 130041, China; ^2^Beijing Super Link Medical Research Institute, Beokomg, Fengtai District, Caihuying No. 58, Room 1102, Beijing 100000, China; ^3^Department of Surgery, School of Medicine, Gansu University of Chinese Medicine, No. 35 Dingxi East Road, Lanzhou, Gansu 730000, China; ^4^Department of Surgery, Xi'an Hospital of Traditional Chinese Medicine, No. 69 Fengcheng, 8th Road, Xi'an, Shanxi 710021, China

## Abstract

The hepatitis A virus cellular receptor 1 (HAVCR1) gene as a sensitive and specific biomarker has been reported in various diseases. Especially, HAVCR1 overexpression promotes the development and progression of several human cancers. Hence, we aimed to detect the effects of HAVCR1 on gastric adenocarcinoma (GAC). We first determined the expression of HAVCR1 in GAC tissues compared with normal gastric tissues based on the Cancer Genome Atlas (TCGA) database using bioinformatics analysis methods. Then, we assessed the biological function of HAVCR1 in GAC cells using quantitative real-time reverse transcription-PCR (qRT-PCR), western blot, cell counting kit-8- (CCK-) 8, colony formation assay, wound healing assay, and transwell assay. Our results showed that HAVCR1 expression was upregulated in GAC tissues and positively associated with poor survival. Loss-of-function analyses indicated that knockdown of HAVCR1 inhibited the proliferation, colony formation, migration, and invasion of GAC cells. Furthermore, reduction of HAVCR1 in GAC cells can decrease the expression of phosphorylated MEK/ERK. These findings suggested that HAVCR1 may represent a potential biomarker for GAC prognosis, as well as a novel therapeutic target for GAC treatment.

## 1. Introduction

Gastric cancer is the fourth most common cancer and the second leading cause of cancer mortality in the world [1]. A large majority (approximately 90%) of gastric cancers are gastric adenocarcinomas [1, 2]. A gastric adenocarcinoma (GAC) arises from the glands of the most superficial layer, or the mucosa, of the stomach, which is related with chronic gastritis, high salt intake, *Helicobactor pylori* infection, smoking, and pernicious anemia [2, 3]. Surgical resection is the standard primary therapy for GAC. However, after surgery, the overall 5-year survival rate remains poor because of the high locoregional and distant recurrence rates [3, 4]. To improve the outcome of surgery, adjuvant or neoadjuvant therapy is frequently used in combination with surgery such as radiotherapy and chemotherapy. However, the addition of radiotherapy and chemotherapy has not been reported to provide any obvious additional benefit [1, 4]. Hence, how to treat GAC effectively has become an urgent task. As target therapy can offer a specific and effective treatment for GAC patients, target therapy has become the research priority of GAC treatment [5, 6]. Therefore, it is critical to choose some effective biomarkers for GAC target therapy.

The hepatitis A virus cellular receptor 1 (HAVCR1) gene maps on the 5q33.2 cytogenetic location, which codes for a type I transmembrane glycoprotein [7, 8]. HAVCR1, also known as human kidney injury molecule-1 (KIM-1) and T-cell immunoglobulin and mucin domain-1 (TIM-1), was initially described in primate kidney cells [9] and is associated with disease susceptibility [10]. HAVCR1 has been reported as a sensitive and specific biomarker for hepatitis [11, 12] and acute kidney injury [13–15]. According to the previous report of Vila et al., HAVCR1 was firstly proven to inhibit cell differentiation and was highly expressed in clear cell renal cell carcinoma [16]. Moreover, emerging evidence suggested that HAVCR1 was correlated with some aggressive tumors such as renal cell carcinoma [7, 17, 18], human colorectal cancer [10], and ovarian clear cell carcinoma [19]. Consequently, HAVCR1 can be considered as an effective biomarker that is related with tumor development and progression.

In this study, we aimed to explore the expression levels of HAVCR1 in GAC and also explore the prognostic significance and relationship between HAVCR1 expression and GAC patients' clinicopathologic characteristics. We also examined the potential function of HAVCR1 in GAC cells.

## 2. Materials and Methods

### 2.1. Database

The dataset of the gene was from TCGA, which includes 375 samples of GAC tissues and 32 samples of normal gastric tissues. The corresponding full-scale clinical information of 306 GAC patients was also obtained from the TCGA database (https://cancergenome.nih.gov/). The data collection processes were in accordance with all laws and regulations.

### 2.2. Cell Culture and Transfection

Human gastric epithelial cell line GES-1 and human gastric cancer cell lines MKN-45 and AGS were purchased from Shanghai Institutes for Biological Sciences (Shanghai, China). Cells were cultured in RPMI-1640 medium supplemented with 10% fetal bovine serum (FBS), 100 U/ml penicillin, and 0.1 mg/ml streptomycin at 37°C in a humid incubator containing 5% CO_2_.

Cells were transfected using Lipofectamine 2000 (Invitrogen Life Technologies, Karlsruhe, Germany) according to the manufacturer's protocol. Two types of siRNAs were used to knockdown the HAVCR1 expression. The siRNA sequences of HAVCR1 are as follows: F: 5′-UCC UUG GUG GGA GAU AGA G-3′ for siRNA1 (si-HAVCR1#1), and F: 5′-GAG AAC UCA GGA ACU CUC A-3′ for siRNA2 (si-HAVCR1#2). Meanwhile, nonspecific siRNA was used as a negative control group (si-con). The transfected cells were harvested after 48 h of transfection for the following experiments. Transfection efficiency was measured by qRT-PCR and western blot.

### 2.3. RNA Extraction and qRT-PCR

Total RNA was extracted using the Trizol Reagent (Invitrogen, Grand Island, NY, USA) according to the manufacturer's protocol. Samples were reverse transcribed to cDNA using a PrimeScript RT reagent Kit (Takara, Dalian, China). The mRNA expression of HAVCR1 was determined by real-time PCR using SYBR Green (Takara, Dalian, China). The qRT-PCR cycle conditions were 95°C for 5 min, 40 cycles of 95°C for 30 sec, 60°C for 40 sec, and 72°C for 1 min. The results were quantified and normalized using the 2^−ΔΔ*Ct*^ method relative to GAPDH. Each assay was independently performed three times. The primers were as follows: HAVCR1 forward, 5′-TGG TGG GAG ATA GAG GAA GCA T-3′, and reverse, 5′-GAT CAG CGT TCA GAT CCA GG-3′; GAPDH forward, 5′-GGA GCG AGA TCC CTC CAA AAT-3′, and reverse, 5′-GGC TGT TGT CAT ACT TCT CAT GG-3′.

### 2.4. Western Blot

Cells were harvested and treated with lysis buffer (KeyGEN, Nanjing, China) on ice for 30 min. A BCA kit (KeyGEN, Nanjing, China) was used to quantify protein concentration. Equal amounts of protein (20 *μ*g) were resolved on SDS-PAGE and transferred to a PVDF membrane. Membranes were blocked with 5% nonfat milk for 1 h and incubated overnight at 4°C with primary antibodies (dilution 1 : 1000, Cell Signaling Technology, Inc., Danvers, MA, USA) against MEK (Cat no. 9126), p-MEK (Cat no. 3958), ERK (Cat no. 4695), p-ERK (Cat no. 8544), GAPDH (Cat no. 5174), and HAVCR1 (Cat no. 14971). Subsequently, membranes were washed with TBST and cultured with secondary antibody (anti-rabbit IgG, HRP-conjugated goat anti-rabbit, dilution 1 : 3000, Cat no. 7074, Cell Signaling Technology, Inc., Danvers, MA, USA) for 2 h at room temperature. The samples were detected by an ECL detection system (Thermo Fisher Scientific). And the intensity of the signal on each membrane was quantified by densitometry (Quantity One software, Bio-Rad). GAPDH was used as control. All experiments were repeated at least three times.

### 2.5. Cell Proliferation Assay

The cell proliferation was determined using CCK-8 according to the manufacturer's protocol. The transfected cells were seeded in 96-well plates at a density 1000 cells per well and determined at 24 h, 48 h, 72 h, and 96 h using a CCK-8 kit. The absorbance was measured at 450 nm using a microplate reader (Thermo Fisher Scientific Microplate Reader, Waltham, Massachusetts, USA). Each experiment was performed independently in triplicate.

### 2.6. Colony Formation Assays

The transfected cells were seeded in six-well plates and incubated in medium with 10% FBS that was replaced every 3 or 4 days. After two weeks, cells were fixed with 4% paraformaldehyde for 30 min and stained with 0.1% crystal violet for 30 min (Sigma-Aldrich, St. Louis, MO, USA). Visible colonies were counted and imaged. Each sample was determined independently at three times.

### 2.7. Wound Healing Assay

The transfected cells were cultured on six-well plates at 37°C in a humid incubator with 5% CO_2_ for 24 h, resulting in a monolayer at more than 90% confluence. Then monolayer cells were scraped with a 200 *μ*l standard pipette tip to generate a wound. The cells were washed with fresh medium to remove floating cells. Subsequently, the spread of the wound closure was recorded after 24 h of incubation under an Olympus BX51 microscope (Olympus Corporation, Tokyo, Japan). The experiment was repeated at three times.

### 2.8. Transwell Migration and Invasion Assays

Transwell assays were carried out in 24-well plates using a transwell chamber with/without Matrigel (pore size 8 *μ*m, BD Biosciences, Lake Franklin, New Jersey, USA). In the migration assay, cells were suspended in serum-free RPMI 1640 medium and added to the upper chamber at 1 × 10^5^ cells/transwell. In the invasion assay, cells suspended in serum-free RPMI 1640 medium were seeded in the upper chamber coated with Matrigel at 1 × 10^5^ cells/transwell. In each assay, the lower chamber was filled with 10% FBS RPMI 1640 medium as a chemoattractant. After 24 hours of incubation, the upper surfaces of the transwells were wiped with cotton swabs. The inserts were fixed with paraformaldehyde for 30 min, stained with 0.1% crystal violet for 20 min, and then washed with PBS. Next, the migrating and invading cells were counted and photographed under an Olympus BX51 microscope (Olympus Corp., Shinjuku, Tokyo, Japan) at 200x magnification over five random fields in each well. The experiment was repeated three times.

### 2.9. Statistical Analysis

All values are expressed as the mean ± standard deviation (SD) and the data were analyzed by SPSS Statistics software (version 22.0, Chicago, IL, USA). The relationship between HAVCR1 expression and clinical characteristics was determined by a Chi-square test and one-way ANOVA. The Kaplan-Meier method and the log-rank test were used to generate overall survival curves. The COX regression analysis was used to detect whether HAVCR1 is an independent risk factor for prognosis in GAC cancer. Student's *t*-test was used to measure significant differences between two groups. Differences were considered significant for *p* < 0.05.

## 3. Results

### 3.1. HAVCR1 Expression Was Upregulated in GAC and Correlated with Poor Prognosis

**Figure 1:**
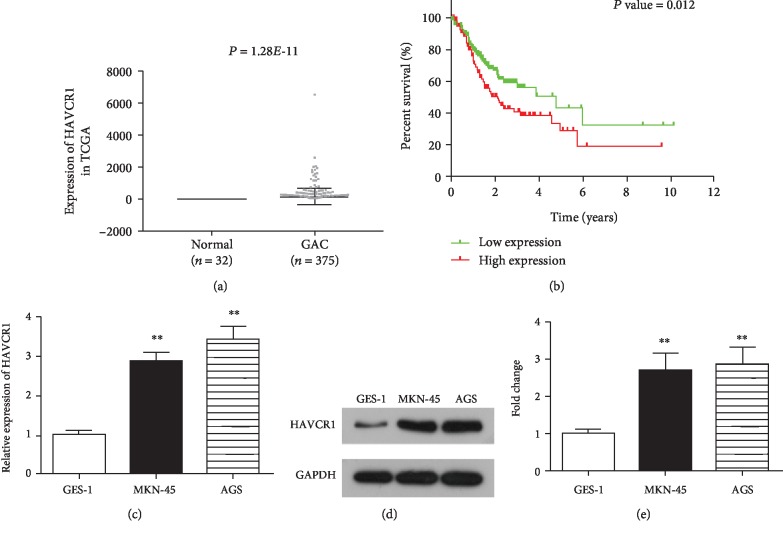
HAVCR1 was upregulated in GAC tissues and cell lines, and high HAVCR1 expression was related with poor prognosis. (a) HAVCR1 expression was upregulated in GAC tissues (*n* = 375) compared with normal gastric tissues (*n* = 32). (b) Kaplan-Meier's survival analysis showed that the high regulation of HAVCR1 had a close relationship with the poor outcome of GAC patients, *p* = 0.012. (c) QRT-PCR reveals the expression levels of HAVCR1 in GAC cells (MKN-45 and AGS) and in a normal gastric cell line (GES-1). (d) Western blot analysis was performed to detect the expression of HAVCR1. (e) The gray value of protein bands was quantified. ^∗∗^*p* < 0.01 versus GES-1 cells.

To identify the potential role of HAVCR1 in GAC, we first assessed HAVCR1 expression in 375 GAC tissues and 32 normal gastric tissues based on The Cancer Genome Atlas (TCGA, https://cancergenome.nih.gov/) database. We found that *HAVCR1* expression was obviously upregulated in GAC tissues (*n* = 375) compared with normal tissues (*n* = 32; *p* < 0.01, Figure 1(a)). Moreover, a Chi-square test revealed that HAVCR1 expression was positively correlated with death (*p* = 0.002, Table 1).

In addition, after excluding patients without follow-up, 306 GAC patients remained and their full-scale clinical information was extracted from TCGA database. The median expression value was used as a cutoff point, and the GAC patients were divided into two groups: the HAVCR1-high group (>median value; *n* = 153) and the HAVCR1-low group (<median value; *n* = 153). The data were analyzed by the Kaplan-Meier method with a log-rank test, and thus overall survival curves were plotted. As shown in Figure 1(b), compared with the HAVCR1-low group, the HAVCR1-high group showed poor overall survival (*p* = 0.012).

Moreover, the prognostic value of HAVCR1 in GAC patients was evaluated by the Cox regression analysis. As shown in Table 2, univariate analysis showed that HAVCR1 expression (*p* = 0.013, HR = 1.58), clinical-stage (*p* = 0.001, HR = 1.883), pathologic-N (*p* = 0.005, HR = 1.873), and age (*p* = 0.011, HR = 1.697) were significantly related to overall survival in GAC patients. Multivariate analysis indicated that HAVCR1 expression (*p* = 0.008, HR = 1.633) and age (*p* = 0.002, HR = 1.954) could be regarded as independent risk factors for overall survival in GAC patients. Collectively, results showed that HAVCR1 might be a potential biomarker and prognostic predictor for GAC.

### 3.2. HAVCR1 Expression Was Upregulated in GAC Cells

**Figure 2:**
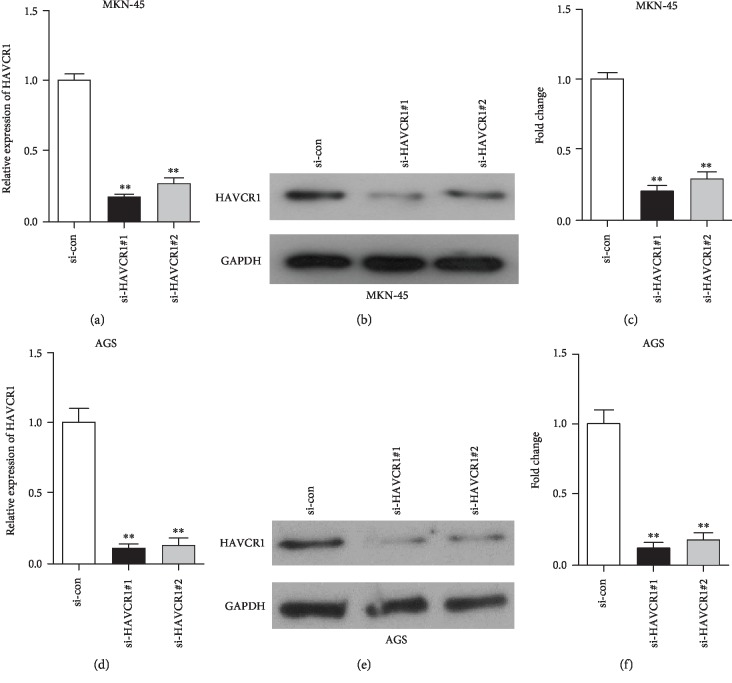
HAVCR1 expression was significantly decreased in MKN-45 and AGS cells using siRNA strategy. (a) QRT-PCR was used to assess the HAVCR1 mRNA expression in MKN-45 cells after si-HAVCR1#1 and si-HAVCR1#2 transfection, respectively, ^∗∗^*p* < 0.01 versus the si-con group. (b and c) The HAVCR1 protein level was evaluated using MKN-45 cells that were transfected with si-HAVCR1#1 or si-HAVCR1#2. The gray value of the protein bands was quantified. ^∗∗^*p* < 0.01 versus the si-con group. (d) QRT-PCR analysis showed the silencing efficiency of si-HAVCR1#1 and si-HAVCR1#2 in AGS cells, ^∗∗^*p* < 0.01 versus the si-con group. (e and f) The expression of HAVCR1 in AGS cells was detected by western blot and quantified, ^∗∗^*p* < 0.01 versus the si-con group.

To investigate the effect of HAVCR1 on GAC cells, we first determined the expression of HAVCR1 in different GAC cell lines using quantitative real-time reverse transcription-PCR (qRT-PCR). As shown in Figure 1(c), when compared with the normal gastric cell line (GES-1), HAVCR1 expression was markedly upregulated in GAC cell lines (MKN-45 and AGS), especially the AGS cell line. Moreover, western blotting analysis exhibited that the protein expression of HAVCR1 in MKN-45 and AGS cells was higher than that of GES-1 cells (*p* < 0.01, Figures 1(d) and 1(e)). These results confirmed that HAVCR1 expression was highly regulated in GAC cell lines. In order to examine the effects of HAVCR1 in GAC cells, we first knocked down the expression of HAVCR1 using siRNA strategy. QRT-PCR and western blot methods were conducted to detect the silencing efficiency in MKN-45 and AGS cells (*p* < 0.01, Figure 2).

### 3.3. Downregulation of HAVCR1 Impaired the Proliferation and Colony Formation of GAC Cells

**Figure 3:**
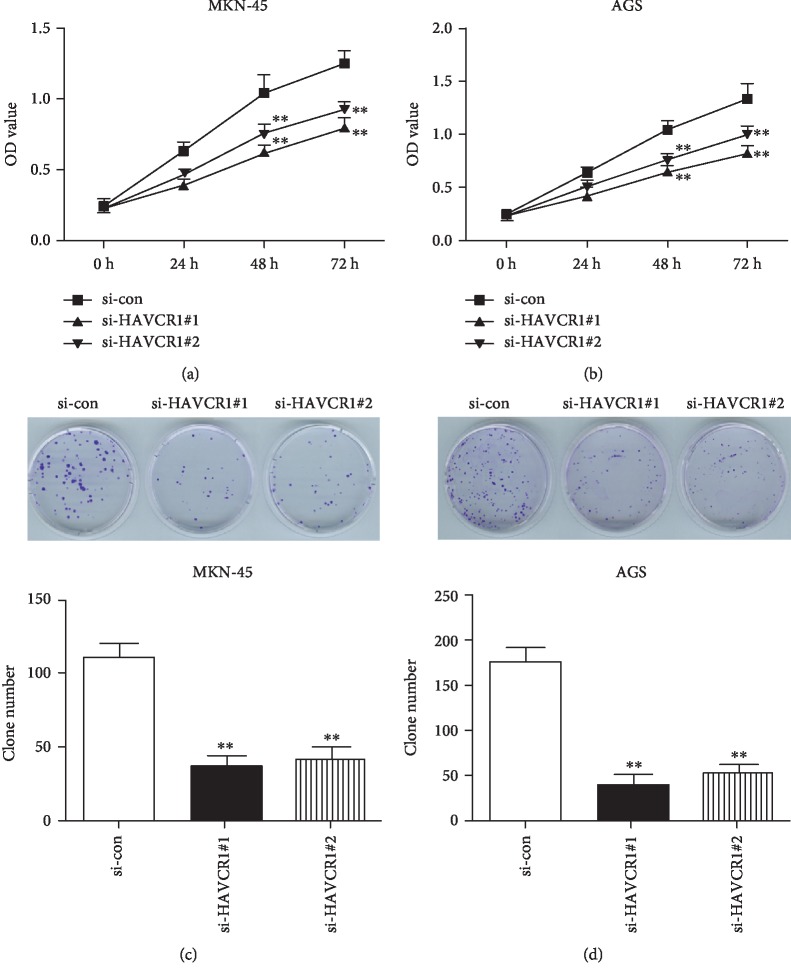
Downregulation of HAVCR1 impaired the proliferation and colony formation of GAC cells. (a and b) A CCK-8 assay was used to measure the proliferation of MKN-45 and AGS cells. (c and d) A colony formation assay was conducted in MKN-45 and AGS cells to further determine the impact of HAVCR1 deficiency on clonogenic capability. And the number of clones was counted. ^∗∗^*p* < 0.01 versus the si-con group.

To explore the effect of HAVCR1 on proliferative and clonogenic abilities of GAC cells, CCK-8 and colony formation assays were performed in MKN-45 and AGS cells. In the CCK-8 assay, the proliferation of MKN-45 and AGS, which were transfected with si-HAVCR1#1 and si-HAVCR1#2, showed an obvious decrease when compared with the negative control group (si-con) (*p* < 0.01, Figures 3(a) and 3(b)). The same trends were also observed in the colony formation assay. As shown in Figures 3(c) and 3(d), the number of clones in the si-HAVCR1#1 and si-HAVCR1#2 groups was significantly decreased relative to the si-con group in two GAC cell lines, including MKN-45 and AGS (*p* < 0.01). Therefore, these observations disclosed that knockdown of HAVCR1 played an inhibitory role in the proliferation and colony formation of GAC cells.

### 3.4. Reduction of HAVCR1 Attenuated the Migration and Invasion of GAC Cells

**Figure 4:**
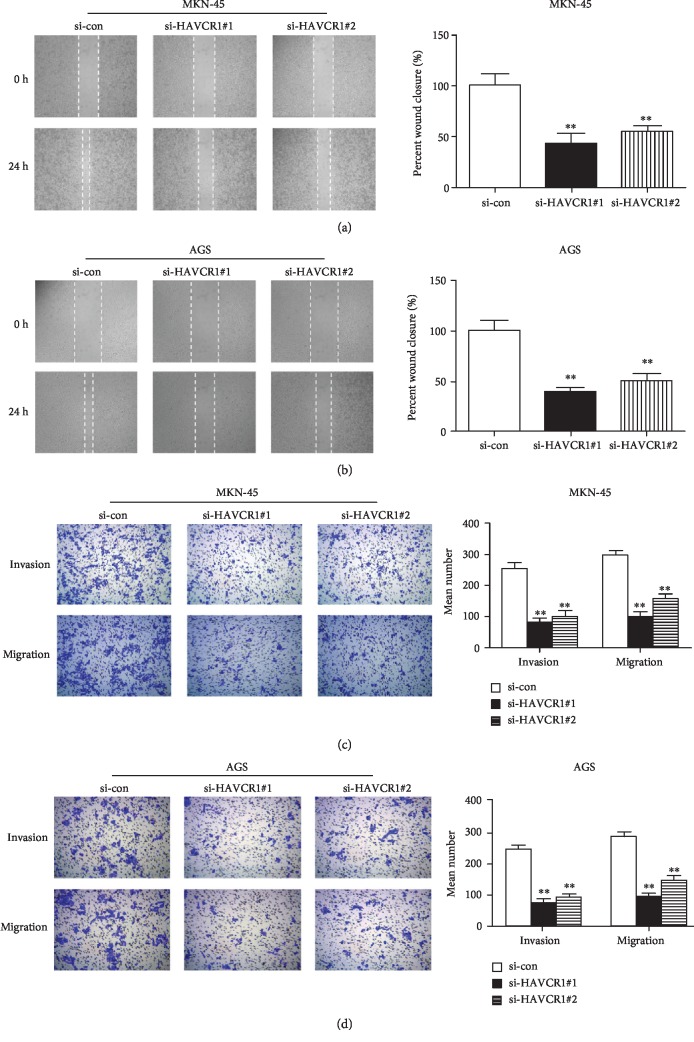
Reduction of HAVCR1 attenuated the migration and invasion of GAC cells. A wound healing assay was implemented to assess the migration of (a) MKN-45 cells and (b) AGS cells. And the distance of the wound was also quantified. (c and d) The migration and invasion of GAC cells was further detected by transwell assays. ^∗∗^*p* < 0.01 versus the si-con group.

To determine the impact of HAVCR1 on the mobility of GAC cells, a wound healing assay and transwell assays were used for this purpose. For wound-healing analysis in MKN-45 cells, the distances of the wound in the si-HAVCR1#1 and si-HAVCR1#2 groups were larger than that in the si-con group after 24 h of incubation (*p* < 0.01, Figure 4(a)). Consistently, the wound closure of the si-HAVCR1#1 and si-HAVCR1#2 groups in AGS cells was wider than that in the si-con group (*p* < 0.01, Figure 4(b)). Furthermore, transwell assays in MKN-45 and AGS cells indicated that si-HAVCR1#1 and si-HAVCR1#2 transfection markedly repressed the migration and invasion of GAC cells compared with si-con treatment (*p* < 0.01, Figures 4(c) and 4(d)). Taken together, the knockdown of HAVCR1 inhibited the migration and invasion of GAC cells.

### 3.5. HAVCR1 Deficiency Affected the Activation of Extracellular Signal-Regulated Kinase (MEK/ERK) Signaling Pathway

**Figure 5:**
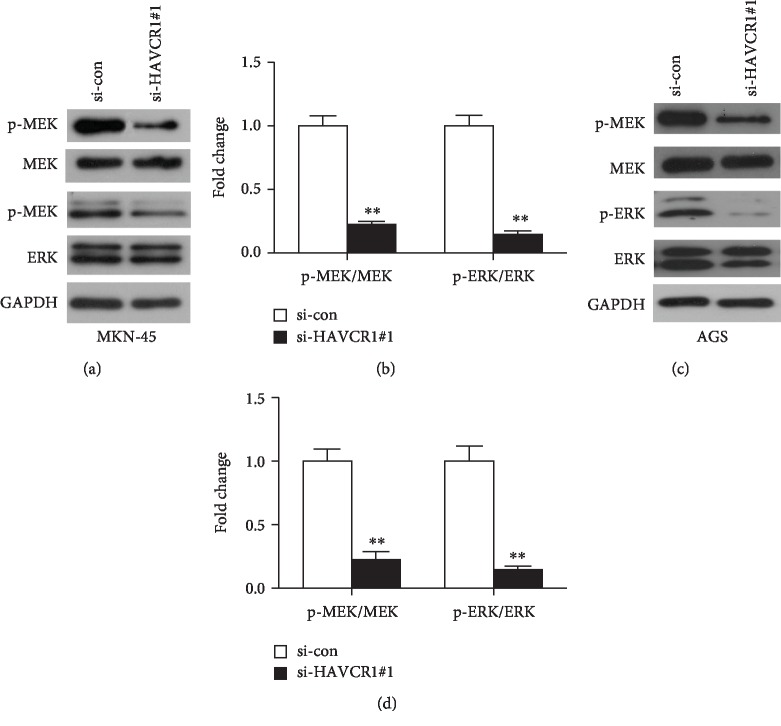
Activation of MEK/ERK signaling pathway was associated with HAVCR1 expression. (a) The MEK/ERK related protein levels were examined by western blot analysis in MKN-45 cells after si-HAVCR1#1 transfection. (b) The gray values of protein bands were quantified. ^∗∗^*p* < 0.01 versus the si-con group. (c) The effects of HAVCR1 deficiency in MEK, p-MEK, ERK, and p-ERK expression levels in AGS cells were measured by western blot. (d) The expression levels of the above proteins were quantified and normalized to GAPDH. One specific siRNA (si-HAVCR1#1) was used as an experimental group because of its higher knockdown efficiency, and nonspecific siRNA was used as a negative control group (si-con). ^∗∗^*p* < 0.01 versus the si-con group.

To investigate the potential mechanism of HAVCR1 on the proliferation, colony formation, migration, and invasion of GAC cells, the ERK signaling cascade, as one of the most frequently dysregulated pathways in human cancer, was determined using western blot. Due to the highest silencing efficiency and more obvious effects of si-HAVCR1#1, the subsequent western blotting experiments were implemented by si-HAVCR1#1. After si-HAVCR1#1 transfection, the expression levels of p-MEK and p-ERK in MKN-45 and AGS cells were significantly downregulated compared to the si-con group (*p* < 0.01, Figures 5(a)–5(d)), whereas the total MEK and ERK expression levels showed no differences between the si-HAVCR1#1 group and the si-con group (*p* < 0.01, Figures 5(a)–5(d)). In summary, HAVCR1 deficiency blocked the activation of the MEK/ERK signaling pathway, thereby affecting the proliferation, colony formation, migration, and invasion of GAC cells.

## 4. Discussion

In this study, we first analyzed the expression levels of HAVCR1 in GAC samples based on TCGA database. We found that HAVCR1 expression was upregulated in GAC tissues compared with the normal gastric tissues and positively related with poor prognosis. Cox analysis showed that HAVCR1 might be an independent predictor for prognosis of GAC patients. Moreover, functional experiments revealed that the downregulation of HAVCR1 suppressed the proliferation, colony formation, migration, and invasion of GAC cells via mediating the activation of the MEK/ERK pathway. Hence, HAVCR1 may be considered as a potential biomarker and prognostic predictor for GAC.

HAVCR1 has been reported to be associated with many diseases such as kidney injury [13–15], infection (hepatitis C virus, hepatitis A virus, and ebolavirus) [11, 12, 20, 21], and autoimmune diseases (allergy, asthma, and systemic lupus erythematosus patients) [22–24]. These studies have shown that HAVCR1 has multiple functions in various diseases. Particularly, the role of HAVCR1 as a receptor in hepatocytes has been widely studied. For instance, HAVCR1 can be a facilitator of hepatitis C virus (HCV) entry, which could stabilize or enhance virus binding to the cell surface membrane and allow the correct virus-receptor positioning for interaction with the main HCV receptors [12]. What's more, HAVCR1 overexpression was observed in renal cell carcinoma [7], human colorectal cancer [10], and ovarian clear cell carcinoma [19], which could promote the development and progression of tumors. Thus, HAVCR1 overexpression may be a diagnostic biomarker for several cancers [10]. According to these findings, we aimed to explore the relationship between HAVCR1 and GAC progression. Based on TCGA database, HAVCR1 expression was upregulated in GAC tissues and correlated with poor survival. Additionally, our experimental results indicated that HAVCR1 deficiency impaired the proliferation, colony formation, migration, and invasion of GAC cells. Therefore, HAVCR1 might be a potential biomarker for GAC prognosis and an effective therapeutic target for GAC treatment.

The ERK signaling cascade is a central MAPK pathway that regulates a variety of cellular processes such as proliferation, differentiation, survival, migration, and invasion [25–27]. ERK can be activated by various oncogenes and extracellular stimuli, and MEK acts as an upstream key protein of ERK [26, 27]. The ERK signaling pathway has been reported to play an important role in gastric cancer cell growth and metastasis [28–30]. More importantly, Zhang and Cai indicated that HAVCR1 can mediate renal epithelial cell repair through the ERK/MAPK signaling pathway [31]. Hence, in this study, we detected the effect of the downregulation of HAVCR1 on the MEK/ERK pathway in AGS cells. The phosphorylation of MEK and ERK expression levels showed an obvious decrease after the knockdown of HAVCR1. Therefore, we guessed that the effects of HAVCR1 on proliferation, colony formation, migration, and invasion of GAC cells might be associated with the MEK/ERK pathway.

## 5. Conclusions

Our data uncovered that HAVCR1 expression was upregulated in GAC tissues and cell lines, and its high regulation was linked with the poor outcome of GAC patients. Moreover, loss-of-function experiments revealed that the downregulation of HAVCR1 played negative effects on the proliferation, colony formation, migration, and invasion of MKN-45 and AGS cells. The MEK/ERK-related proteins were also decreased after HAVCR1 inhibition. To sum up, HAVCR1 may be a potential biomarker for GAC prognosis, as well as a novel therapeutic target for GAC treatment. Of course, further studies are needed to validate these findings and to clarify the detailed mechanism for the promoting role of HAVCR1 on GAC.

## Figures and Tables

**Table 1:** Correlation between HAVCR1 expression and clinicopathological characteristics of GAC patients.

Characteristics	Expression of HAVCR1		*p* value
	Low	High	
*Age*			0.067
<60	42	57	
≥60	111	96	
*Gender*			0.813
Female	56	56	
Male	95	97	
*Grade*			0.478
G1+G2	60	54	
G3	93	99	
*Clinical stage*			0.359
I+II	74	66	
III+IV	79	87	
*Pathologic-T*			0.290
T1+T2	34	42	
T3+T4	119	111	
*Pathologic-N*			0.711
N0	49	46	
N1	104	107	
*Pathologic-M*			0.644
M0	144	142	
M1	9	11	
*Death*			0.002^∗^
Yes	49	75	
No	104	78	

T: tumor status; N: regional lymph node status; M: metastasis status. ^∗^Significant correlation.

**Table 2:** Univariate and multivariate analysis of clinic pathologic factors for overall survival in GAC patients.

Variables	Univariate analysis			Multivariate analysis		
	*p* value	HR	95% CI	*p* value	HR	95% CI
HAVCR1 expression (high/low)	0.013^∗^	1.580	1.101-2.265	0.008^∗^	1.633	1.134-2.353
Clinical stage (I+II/III+IV)	0.001^∗^	1.883	1.292-2.745	0.092	1.534	0.933-2.523
Pathologic-T (T1+T2/T3+T4)	0.064	1.529	0.975-2.398			
Pathologic-M (M0/M1)	0.126	1.659	0.868-3.173			
Pathologic-N (N0/N1+N2+N3)	0.005^∗^	1.873	1.207-2.906	0.225	1.431	0.802-2.553
Age (<60/≥60)	0.011^∗^	1.697	1.128-2.551	0.002^∗^	1.954	1.292-2.955
Gender (female/male)	0.083	1.405	0.956-2.065			
Grade (G1+G2/G3)	0.124	1.340	0.923-1.946			

HR: hazard ratio; CI: confidence interval. ^∗^Significant correlation.

## Data Availability

The data used to support the findings of this study are available from the corresponding author upon request
